# Feasibility investigation of uniportal video-assisted thoracoscopic anatomical lung resection for pulmonary sequestration

**DOI:** 10.1186/s13019-020-01126-x

**Published:** 2020-05-13

**Authors:** Yungang Sun, Feng Shao, Qiang Zhang, Zhao Wang

**Affiliations:** 1 Department of Thoracic Surgery, Nanjing Chest Hospital, Nanjing Brain Hospital Affiliated to Nanjing Medical University, 264 Guangzhou Road, Nanjing, 210029 China; 2 Pulmonary Nodule Diagnosis and Treatment Research Center, Nanjing Medical University, Nanjing, China

**Keywords:** Uniportal video-assisted thoracic surgery, Pulmonary sequestration, Mini-invasive technique

## Abstract

**Background:**

Uniportal video-assisted thoracic surgery (UVATS) technique has been increasingly used for many thoracic diseases. Whether UVATS has equivalent or better perioperative outcomes for pulmonary sequestration (PS) patients remains controversial. Our study aimed to evaluate the feasibility of UVATS in anatomical lung resection for pulmonary sequestration.

**Methods:**

A total of 24 patients with PS including fifteen males and nine females with the mean age of 40 (range, 18–65) years old, who had received completely UVATS anatomical lung resection for PS in Nanjing Chest Hospital between January 2016 and December 2018 were retrospectively reviewed. Related clinical data were retrieved from hospital records and analyzed.

**Results:**

All 24 patients had been treated with the UAVTS approach successfully without aberrant artery ruptured or massive hemorrhage, and no patients died during the perioperative period. Overall mean surgery time was 102 mins (range, 55–150 min), the mean blood loss was 94 ml (range, 10-300 ml), the mean days of chest tube maintained were 4 days (range,1-10 days), and the mean postoperative hospitalization days was 6 days (range,2-11 days). All patients were cured, without cough, fever, hemoptysis, and so on, associated with PS, occurring during the average follow-up of 17 months (range, 3-35 months).

**Conclusions:**

Our preliminary results revealed that anatomical lung resection by UVATS is a safe and feasible mini-invasive technique for PS patients, which might be associated with less postoperative pain, reduced paresthesia, better cosmetic results, and faster recovery.

## Background

Pulmonary sequestration (PS) is a rare congenital malformation of the lung, in which an island of the lung parenchyma lacks a normal communication with the tracheobronchial tree and receives arterial blood supply from an aberrant branch of systemic circulation rather than the pulmonary circulation, whereas the venous connection is almost normal via pulmonary veins or systemic circulation [1]. As one of the most common types of a congenital abnormality of the lower respiratory tract, PS accounted for 0.15–6.4% of all congenital pulmonary malformations [2]. The theory of the embryologic basis of PS is still unclear, and one of the most widely accepted explanation is that PS results from the formation of an accessory lung bud caudal to the normal lung bud [3, 4]. Generally, the anomalous arterial blood supply usually comes from single or less multiple branches of the thoracic aorta, followed by the abdominal aorta and intercostal artery [5]. According to the presence or absence of independent visceral pleura covering at the normal lung parenchyma, PS can be divided into two types: intralobar and extralobar pulmonary sequestration [6]. Intralobar pulmonary sequestration (ILS) is often found in the lower lobes, especially in the left lower lobe. However, extralobar pulmonary sequestration (ELS) can appear in many spaces, common in the lower lungs and the spine, occasionally in mediastinum, diaphragm, abdomen. PS can often cause respiratory infection, which will lead to a mass of complications, from common pneumonia, emphysema, and lung abscesses to severe hemoptysis, congestive heart failure, and even malignant transformation [4, 6, 7]. Therefore, this means that it is very important to diagnose the PS correctly. Digital subtraction angiography (DSA) once considered being the gold standard for the diagnosis of PS, because it clearly shows the aberrant vessels. So far, with the advent of non-invasive techniques, such as computed tomography angiography (CTA) has become the most commonly used clinical practice in the diagnosis of PS, DSA has fallen out of favor, because of its ingenuity.

Currently, surgical resection is the best way to treat PS regardless of symptoms, to resolve lesions, make a definite diagnosis and prevent future complications [8]. Previously, many surgeons have demonstrated the feasibility of thoracotomy in treating ILS and ELS [6, 9]. With the development of VATS surgical techniques and sophisticated instruments, VATS has increasingly become the preferred operative procedure in the treatment of PS, which is associated with the potential advantages including less postoperative pain, better cosmetic results, and faster recovery [10–14]. The pursuit for mini-invasive keeps promoting the evolution of VATS from traditional multiple-port to single-port for most VATS resections, and with the progress of VATS experience, UVATS has become technically feasible to perform lung resections. When compared with traditional multiple-port VATS, UVATS was found to result in equivalent perioperative outcomes and reduced postoperative pain and discomfort [15]. Despite these advancements and the popularization of the UVATS technique, lung resection for PS remains a prohibited area. The technical restrictions come from both dense pleural adhesions and the unknown abnormal arteries. Therefore, UVATS in the treatment of PS had seldom been reported [16–19]. Nevertheless, as far as we know, this is the largest number of patients that report the use of UVATS in the treatment for PS.

Herein, the present study aimed to share some points for dealing with the abnormal arteries and discussing the safety and feasibility of lung resection by UVATS for patients with PS.

## Methods

### Patients selection

A total of 24 patients with PS including fifteen males and nine females with the mean age of 40 (range, 18–65) years old, who had received completely UVATS anatomical lung resection of PS in Nanjing Chest Hospital between January 2016 and December 2018 were retrospectively reviewed (Table 1). All the lesions located in the lower lobe with 1–3 aberrant arteries. Four (16.7%) in the right lower lobe, 20 (83.3%) in the left lower lobe. This retrospective study was approved by Nanjing Chest Hospital Review Board and the Ethics Committee.

**Table 1:** Clinical data

Characteristics	Value
Gender	
Male	15 (62.5%)
Female	9 (37.5%)
Age(y)	
Mean	40.54 ± 4.49
Range	18–65
Preoperative symptoms	
Yes	21 (87.5%)
No	3 (12.5%)
Types	
ILS	21 (87.5%)
ELS	3 (12.5%)
Locations	
Left lower lobe	20 (83.3%)
Right lower lobe	4 (16.7%)
Origin of aberrant artery	
Thoracic aorta	20 (83.3%)
Abdominal aorta	3 (12.5%)
Other	1 (4.2%)
Number of aberrant artery	
1	20 (83.3%)
2	3 (12.5%)
3	1 (4.2%)
Maximum caliber of aberrant artery (mm)	
Mean	7.50 ± 3.65
Range	4–20

*ILS* Intralobar pulmonary sequestration; *ELS* Extralobar pulmonary sequestration.

Patients were selected inclusion in the study based on the following criteria: (i) good cardiopulmonary function without surgical contraindications; (ii) no history of surgery on the chest; (iii) preoperative symptoms were controlled smoothly; (iv) all operations were performed under UVATS and completed by prioritizing the division of the aberrant artery; (v) all patients were written informed consent before operation and approval from the institutional review board at our hospital. Exclusion criteria for this study as follows: (i) failure to establish one lung ventilation; (ii) previous history of combined thoracotomy; (iii) diffuse dense adhesions cannot use UVATS.

### Preoperative work-up

The preoperative workup for PS mainly includes two aspects, one was to preoperative diagnosis and then guide surgery, and the other was to control the symptoms of infection. In our institution, the computed tomography angiography (CTA) was suspected for PS, as CTA images could identify the aberrant artery from systemic circulation and guide surgical planning. If necessary, digital subtraction angiography (DSA) or magnetic resonance imaging (MRI) should be performed.

Patients with respiratory symptoms were candidates for initial antibiotic treatment, in order to control the preoperative inflammation and increase the safety of surgery. Of the 24 patients in this study, the main symptoms were expectoration (11 cases, 45.8%), followed by cough (5 cases, 20.8%), fever (2 cases, 8.3%), chest pain (2 cases, 8.3%), and hemoptysis (1 case, 4.3%). Three (12.5%) patients were asymptomatic and did not need preoperative antibiotics (T**able** **2**).

**Table 2:** Preoperative symptoms

Main symptoms	N(%)
expectoration	11 (45.8%)
cough	5 (20.8%)
fever	2 (8.3%)
chest pain	2 (8.3%)
hemoptysis	1 (4.2%)
asymptom	3 (12.5%)

Lobectomy of the lung lesion located was performed for patients with ILS, however, mass excision of the sequestered lung tissue was performed for all ELS patients. Caliber and number of aberrant arteries were not considered as contraindications to UVATS.

### Surgical technique

All Patients were placed in a lateral decubitus with the arm in the swimming position, which was raised for wide intercostal space, and the operations were performed after a double-lumen endotracheal being inserted. For UVATS, only an approximately 3 cm single incision was made at the fourth or fifth intercostal space of the anterior axillary line with the inserted plastic wound protector (Johnson, New Brunswick, NJ, USA). The vision was obtained through a thirty-degree, 10 mm video-thoracoscope (Karl Storz, Tuttlingen, Germany).

Before any lung resection was performed, we needed to exclude any significant adhesions in the whole chest cavity carefully. Usually, we held the gauze with oval forceps and bluntly pushed away the adhesions between the visceral pleura and the parietal pleura and alternately used the electrocoagulation hook for sharp separation without using any other special devices. According to the preoperative imaging, proceeded to separate the inferior pulmonary ligament, looking carefully for any aberrant artery, where it was always located in. Hereafter, remove the aberrant arteries depends on some methods. Drawing on our experience, the smaller aberrant artery was first ligated proximally using a non-absorbable suture, then proximally and distally clipped with Hemo-o-Lock clips, and then finally was cut with scissors. However, the bigger aberrant artery was also first ligated proximally using a non-absorbablenot-resorbable suture and then divided using a vascular endostapler (Ethicon Endo-Surgery, Inc). Due to the high pressure of aberrant arteries, suture ligation was very necessary to reduce the risk of bleeding. After ensuring all aberrant arteries were divided, which gave more security to avoid bleeding complications, then the lung resection should be performed. For patients with ILS, a routine dissection that dividing the lobar vein, bronchus, and artery in an order, was a completed lobectomy using the standard uniportal technique [20]. For patients with ELS, determining and dissecting the vein drainage, which was passed through the accessory fissure between the lower lobe and the lesion, and then separated the fissure between the lesion and lower lobe with endostapler. At the end of the operation, the arterial stump was covered with fibrin glue and a single 20th chest tube was used to drain the pleural space via the uniport.

### Postoperative management and follow-up

After surgery, the patient was transferred to the normal surgical ward and with ECG monitoring for one night. And then early removal of the urine tube and the ECG monitor, allowing patients to mobilize early, promoting lung recruitment and pleural effusion, which was involved as part of rapid recovery. When there was no air-leakage and fluid drainage was less than 200 ml within 24 h, the chest drain was removed. Patients were usually discharged the day after removal of the chest drain and were followed up in the outpatient clinic. The presence of respiratory infection symptoms, such as cough, fever, hemoptysis, and so on, were assessed.

### Statistical analyses

All data were analyzed using SPSS version 21.0 (SPSS Inc., Chicago, IL, USA). The continuous data were presented as mean ± standard deviation. The categorical variables were presented as numbers.

## Results

All the surgeries were carried out using the UAVTS approach successfully with no intraoperative conversions or complications. Also, no aberrant artery ruptured or massive hemorrhage occurred.

21(87.5%) ILS were received lobectomy and 3(12.5%) ELS were received mass excision. Overall mean surgery time was 102 mins (range, 55–150 min), the mean blood loss was 94 ml (range, 10-300 ml), the mean days of chest tube maintained were 4 days (range,1-10 days), and the mean postoperative hospitalization days was 6 days (range,2-11 days) (Table 3). No patients died during the perioperative period and only one patient developed early postoperative complications: asymptomatic pneumothorax. (Table 4).

**Table 3:** Operation outcomes

Variable	Value
Pleural adhesion	
Yes	15 (62.5%)
No	9 (37.5%)
Surgical resection	
Lobectomy	21 (87.5%)
Mass excision	3 (12.5%)
Surgery time (min)	
Mean	102
Range	55–150
Blood loss (ml)	
Mean	94
Range	10-300
First day drainage volume (ml)	
Mean	222
Range	10–450
Drainage tube maintain(d)	
Mean	4
Range	1-10
postoperative hospital stay(d)	
Mean	6
Range	2-11
Follow-up time (months)	
Mean	17
Range	3–35

**Table 4:** Complications

Variable	Value
Intraoperative complications	
Pulmonary congestion	1 (4.2%)
Aberrant artery rupture	0
Massive hemorrhage	0
Postoperative complications	
Pneumothorax	1 (4.2%)
Pulmonary infection	0
Bronchopleural fistula	0
Pleural effusion	0
Arrhythmia	0

All patients were diagnosed as PS according to postoperative pathology and were cured, without cough, fever, hemoptysis, and so on, occurring during the average follow-up of 17 months (range, 3-35 months). (Table 3).

## Discussion

PS is an uncommon lung tissue that lacks communication with the normal bronchial tree and receives its aberrant artery blood supply from the systemic circulation, most frequently the descending aorta [1]. PS is classified into two main types: ILS and ELS. ILS is the most common variant, accounting for 75–93% of all PS cases [6, 21, 22] and 87.5% in our study. Both two types are more common in lower lobes in the literature [6], and all patients in our study located in lower lobes, especially in the left lower lobe. PS is a congenital and benign disease, but the potential complications are serious, which may include common pneumonia, emphysema, lung abscess, severe hemoptysis, congestive heart failure, and even malignant transformation [23–25]. Therefore, surgical resection of PS is the preferred and definitive treatment of choice after the diagnosis is made.

The conventional surgical procedure is open posterolateral thoracotomy, which is safe and feasible, however, this technique might lead to greater trauma and pain, and patients recover more slowly [3, 26]. More recently many surgeons have confirmed the feasibility of VATS for PS, because of its minimally invasive, pain relief and quick recovery [10–14]. However, surgeons’ pursuit of ultimate minimally invasive goals never stops. At present, UVATS has become an increasingly popular approach for surgical treatment of thoracic diseases, including PS. The potential advantages of UVATS include more intuitive vision, less postoperative pain, reduced paresthesia, and better cosmetic results compared with the conventional multiple-port VATS [27, 28].

Nevertheless, UVATS remains many difficulties, as they still some controversies regarding the safety and feasibility associated with this technique, including small operating space, instruments interfering with each other and fewer entry points for introducing staplers [15]. In addition to the PS patients, repeated infections lead to dense adhesion of the pleural cavity, and more importantly, the aberrant arteries are very concealed and difficult to handle. All of the aforementioned challenges may be the contraindications of the uniportal access for the resection of PS.

In our institute, we have quite a lot of UVATS experience, in addition to conventional lobectomy and segmentectomy, and many complex operations, such as bronchus sleeve lobectomy, double sleeve lobectomy, and trachea resection can be performed, which have overcome some limitations of the UVATS itself. However, to our knowledge, only few previous studies of UVATS in the treatment of PS were published [16–19, 29]. The first challenging step is to separate the presence of extensive adhesions. The following is to make the identification and closure of the aberrant artery, which is a supply from the systemic circulation. In case of failure to safely remove aberrant arteries, it can lead to fatal catastrophic hemorrhage. In this case, it is necessary and relatively safe to follow the principle of “first abnormal blood vessels, posterior pulmonary blood vessels”, priority to closure aberrant arteries.

In this study, anatomical lung resections of PS were carried out using the UAVTS approach successfully with no significant postoperative complications and no perioperative death in all 24 cases. In terms of skills and details during operation, the following conclusions are made: (i) Preoperative preparations must be sufficient, as for patients with symptoms of infection, antibiotics must be used to control the symptoms of infection, because repeated infections not only suggest that patients may have extensive pleural adhesions, but patients in the infected period are prone to postoperative sepsis, it also increases the difficulty of identifying aberrant arteries. In addition, preoperative CTA, if necessary combined with MRI or DSA, to identify the caliber and number of aberrant arteries, is essential for surgical planning. As the principle of “first abnormal blood vessels, posterior pulmonary blood vessels” is followed, it is an indispensable rule to get the basic information of the aberrant artery before surgery. (ii) The importance of a single incision is not negligible, an approximately 3 cm single incision is made in the fifth intercostal space along the anterior axillary line and slightly backward. Because the PS is basically in the lower lung, and the aberrant arteries are usually located in the inferior pulmonary ligament, and the incision is slightly backward to gain better exposure of the aberrant arteries, which can make the vision more intuitive and increase the safety of the operation. (iii) For most patients with PS, pleural adhesions are present, and 16 of our 24 patients had various kinds of adhesions. For pleural adhesions, we should carefully and alternately use the gauze with oval forceps and electrocoagulation hook, as little as possible lung injury and extensive oozing, less lung air leakage, and postoperative drainage, fully loosening adhesions, mainly increase lung freeness, easy to flip, adjust the angle, get a better surgical vision. Of course, thoracotomy or multiple-port VATS is a wise choice when the adhesions are too dense for dissection by UVATS. (iv) Before any lung resection is performed, the most important step is to find out the aberrant arteries. Combined with the preoperative images and clinical experience, the aberrant artery is mainly located in the inferior pulmonary ligament and enters the lung tissue. Note that some uncommon local sources, such as abdominal aorta and intercostal artery, and so on, in this study, most of the aberrant arteries could be identified by preoperative CTA and other tests, without damage to aberrant arteries, the mean intraoperative blood loss was 94 ml (range, 10-300 ml) and the most bleeding patient was 300 ml, mainly due to dense adhesion of the pleural cavity. (v) Drawing on our experience, removal of the aberrant arteries should depend on their caliber and avoid blind clamping, cutting and sewing. The smaller aberrant artery was first ligated proximally using a non-absorbable suture, then proximally and distally clipped with hemo-o-Lock clips, and then finally was cut with scissors. However, the bigger aberrant artery was also first ligated proximally using a non-absorbable suture and then divided using a vascular endostapler. Due to the local infection, lacking of muscle layers and high pressure of aberrant artery, it is easy to rupture and hemorrhage, so suture ligation is very necessary to reduce the risk of bleeding. Following make sure that all aberrant arteries are processed. Sometimes more than one aberrant artery may exist, if there are any omissions, and the pulmonary veins are divided first, it will cause the congestion of the lungs to occupy most of the chest, which will interfere with the surgical field and increase the risk of surgery. In addition, if the aberrant artery branch left unprocessed, it may encounter unpredictable damage that causes the aberrant arteries to retract to the mediastinum or inside the diaphragm causing uncontrollable bleeding. We strongly recommend that for a wider inferior pulmonary ligament, careful dissection must be done to ensure that the isolated aberrant arteries are not missing. Besides the number of aberrant arteries determined should be consistent with the preoperative images, and any suspect aberrant artery should be treated with caution. In our studies, one patient was found to have rapid hyperemia and swelling of the diseased lung after the inferior pulmonary vein was cutting off. Almost half of the chest cavity was occupied, and the anesthesiologist found that there was also oozing in the double-lumen endotracheal. After careful investigation, a small diameter of about 8 mm was presented in the lower part of the inferior pulmonary vein originated from the descending aorta. After the dissection of this aberrant artery, hyperemia and swelling of the diseased lung and oozing in the double-lumen endotracheal were stopped. (vi) For ILS patients, although literature reported that sublobar resection was feasible, we recommend lobectomy to reduce the likelihood of recurrence. We have previously reported the UVATS lobectomy procedure [20]. For ELS patients; only need a mass excision, but because the anatomical location is close to the esophagus, surgery should be careful to avoid injury to the esophagus, if necessary, a stomach tube can be applied to assist operation preoperative or intraoperative. (vii) Last but not least, in the surgical operation surface, especially on the stump of the aberrant arteries, the fibrin glue is covered to reduce the air leakage, exudation and drainage, so that the chest tube can be removed early and the hospital stay can be shortened.

Additionally, the potential limitations of the present study may include a small size of retrospective cases study, and a lack of statistical data analysis with other surgical approaches. In our further study, we would increase the sample size and further investigate the clinical outcomes to confirm the safety and feasibility of UVATS technique for anatomical lung resection of pulmonary sequestration.

## Conclusions

In virtue of the surgical technical advantages, anatomical lung resection for PS patients using the UVATS approach ensured the safety and feasibility of the procedure and might benefit more patients with PS from minimally invasive treatment. Moreover, UVATS approach might be associated with less postoperative pain, reduced paresthesia, and better cosmetic results compared with the conventional thoracotomy or multiple-port VATS approach.

## Supplementary information

**Additional file 1.**

## Data Availability

The datasets used and analyzed during this study are available from the corresponding author on reasonable request.

## Abbreviations

UVATS: Uniportal video-assisted thoracic surgery
PS: Pulmonary sequestration
ILS: Intralobar pulmonary sequestration
ELS: Extralobar pulmonary sequestration
DSA: Digital subtraction angiography
CTA: Computed tomography angiography
MRI: Magnetic resonance imaging
